# A Work-Related Musculoskeletal Disorders (WMSDs) Risk-Assessment System Using a Single-View Pose Estimation Model

**DOI:** 10.3390/ijerph19169803

**Published:** 2022-08-09

**Authors:** Young-Jin Kwon, Do-Hyun Kim, Byung-Chang Son, Kyoung-Ho Choi, Sungbok Kwak, Taehong Kim

**Affiliations:** 1 Intelligent Robotics Research Division, Electronics and Telecommunications Research Institute, Daejeon 34129, Korea; 2 School of Information and Communication Engineering, Chungbuk National University, Cheongju 28644, Korea; 3 Department of Rehabilitation Technology, Korea Nazarene University, Cheonan 31172, Korea; 4 Department of Electronics Engineering, Mokpo National University, Muan 58554, Korea; 5 Advanced Engineering Team, Duckyang Co., Ltd., Suwon 16229, Korea

**Keywords:** WMSDs, RULA, dataset, deep learning, pose estimation

## Abstract

Musculoskeletal disorders are an unavoidable occupational health problem. In particular, workers who perform repetitive tasks onsite in the manufacturing industry suffer from musculoskeletal problems. In this paper, we propose a system that evaluates the posture of workers in the manufacturing industry with single-view 3D human pose-estimation that can estimate the posture in 3D using an RGB camera that can easily acquire the posture of a worker in a complex workplace. The proposed system builds a Duckyang-Auto Worker Health Safety Environment (DyWHSE), a manufacturing-industry-specific dataset, to estimate the wrist pose evaluated by the Rapid Limb Upper Assessment (RULA). Additionally, we evaluate the quality of the built DyWHSE dataset using the Human3.6M dataset, and the applicability of the proposed system is verified by comparing it with the evaluation results of the experts. The proposed system provides quantitative assessment guidance for working posture risk assessment, assisting the continuous posture assessment of workers.

## 1. Introduction

Work-related musculoskeletal disorders (WMSDs) are an unavoidable occupational health problem for workers and significantly affect their quality of life. Damage caused by exposure to problematic work environments can negatively affect the employment potential of workers [1], and this is emerging as a significant social problem in that it can lead to high costs for businesses and society as a whole [2]. The Eurostat Labour Force Survey ad hoc module “Accidents at work and other work-related health problems”, reported that 60% of the population had musculoskeletal disorders [3]. The World Health Organization estimated that approximately 1.71 billion people worldwide suffer from musculoskeletal disorders and predicts that the incidence of these disorders will continue to increase [1].

To solve this problem, the industry sector has endeavored to prevent musculoskeletal disorders by developing and adopting various assessment methods with the goal of improving work conditions, such as the workloads, postures, work time, and task-performing methods, by analyzing risk factors of the workplace. Ergonomic assessment tools developed to analyze the risk factors of musculoskeletal disorders include the Ovako Working-posture Analysis System (OWAS) [4], rapid upper-limb assessment (RULA) [5], and rapid entire body assessment (REBA) [6], which are typically applied in industries where whole-body postural loads are assessed [7]. The OWAS was developed in 1977 to assess the improper working postures of workers in the steel industry, where heavy materials are often handled. This assessment tool examines the work-performing postures of the waist, arms, and legs and the load and force of the work materials handled, but it is fundamentally limited for the analyses of the posture of the whole body, effectively because it simplifies one’s posture significantly. The RULA method was developed in 1993 to analyze work in the manufacturing industry and focuses on the analysis of upper limbs, such as the shoulders, elbows, wrists, and neck. Additionally, REBA was developed in 2000 to perform an analysis of the service sector, where workers assume various unpredictable postures, such as the upper-limb postures of nurses in patient care. These assessment tools capture the snapshot of work pose and code the postures defined for each body part according to visual measurements, and they are capable of quickly and easily analyzing postures without disturbing the worker [8,9].

As sensors and image-processing technology have advanced substantially, many studies have been conducted to improve visual measurement methods quantitatively [10,11,12,13,14,15,16,17,18,19,20,21,22,23,24,25,26,27,28]. In one of these methods, wireless Inertial Measurement Units (IMUs) sensors can be attached to the worker’s body to obtain movement data [10,11,12,13,17,18,19,26]. However, wearable devices are inconvenient because they must be worn by workers in all processes that require assessments [14,29], and a calibration procedure for the sensors is needed to maintain the accuracy [10,13,18,19].

Research is actively underway to overcome these drawbacks of wearable devices by using cameras, which are noncontact sensors. Furthermore, as keypoint datasets have been released for image-based human pose-estimation and its scope has been expanded from two dimensions [30,31] to three dimensions [32,33,34], various methods based on depth [14,15,16], multiple-view [20,21], and single-view [22,23,24,25] human pose-estimation models have been studied.

An RGB-D camera represented as Microsoft’s Kinect can obtain depth information in addition to the color information of the RGB camera. In previous studies, this technology was used to capture human activity recognition and assess postures [14,15,16,35,36,37,38,39], and it is also used to build datasets [40].

Although motion-capture systems can provide accurate data in ergonomic risk assessments [41], single-view three-dimensional (3D) pose estimation is a method of estimating 3D human pose from the input of a single RGB image. Because of a difficulty to attach motion-capture systems to manufacturing workers, single-view 3D pose estimation is attractive for acquiring and analyzing human motions with a simple image-capturing system. Therefore, we present a single-view 3D pose-estimation model that allows worker images to be captured more simply in complex and various onsite environments in the manufacturing industry.

In addition, previous studies [27,28,29] have a limitation that they excluded wrist analysis in accessing a worker’s posture with RULA. On the contrary, we construct a dataset from images obtained in onsite environments in the manufacturing industry to infer a worker’s wrist posture in a conventional 3D pose-estimation model. It is used in experiments to verify that the model’s estimation performance has been improved so that it can be used for all assessment items, including wrist postures in RULA.

This paper is structured as follows. In Section 2, we summarize the requirements of the OWAS, RULA, and REBA, which are ergonomic precision-assessment tools. We use OpenPose [42,43,44,45,46] to analyze the postures of workers obtained from an automobile cockpit module assembly site and derive problems. Section 3 introduces a method of building a dataset from images obtained in an uncontrolled onsite environment, and Section 4 presents a performance comparison between the model trained using the in-house-built dataset and the assessments of ergonomics experts. Section 5 describes the pose-estimation model and posture evaluation method. Section 6 discusses the validity and importance of the results, and Section 7 presents the conclusions and suggests directions for future research.

## 2. Requirements of Ergonomics Assessment Tools

Table 1 summarizes the classifiable posture parameters and risk levels of the OWAS, RULA, and REBA, which are typical assessment tools for the risk-factor analysis of musculoskeletal disorders of workers, where the number in the parentheses indicates the number of postures that can be classified in each body part. We can observe that the OWAS is more specialized for lower-limb analysis than RULA and REBA. RULA and REBA classify upper and lower arms to assess arm postures and investigate wrist postures; thus, they considerately evaluate the upper-limb pose.

**Figure 1:**
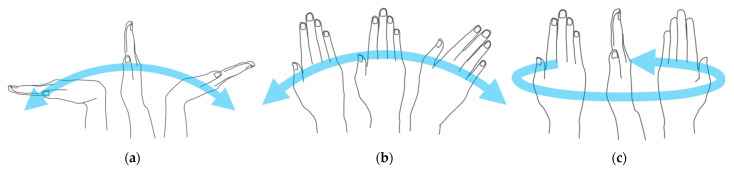
Located wrist positions assessment items options in RULA and REBA: (**a**) locate wrist position; (**b**) lateral deviation; and (**c**) twisted.

Although RULA and REBA are specialized for upper-limb analysis and wrist postures are included as illustrated in Figure 1, previous studies [27,28,29] based on RGB images excluded wrist postures in their methods despite evaluating the worker’s posture with RULA.

To address the worker’s postures, including their hand posture, and examine whether environmental effects occurred in the representative images of the targeted assembly process sites in the manufacturing industry, we used a representative human pose-estimation model. OpenPose can perceive the pose of the face, hand, and other body parts of multiple persons via a bottom-up approach [42,43,44,45,46]. The OpenPose 1.7.0 Whole Body Python API was used for the experiment, with the Body_25 and hand models.

**Figure 2:**
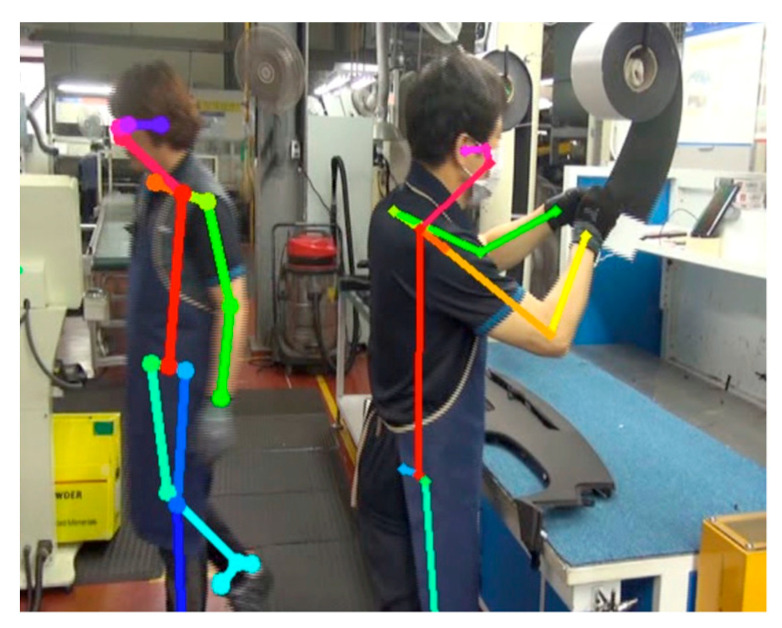
Estimated body pose-estimation results by OpenPose.

**Figure 3:**
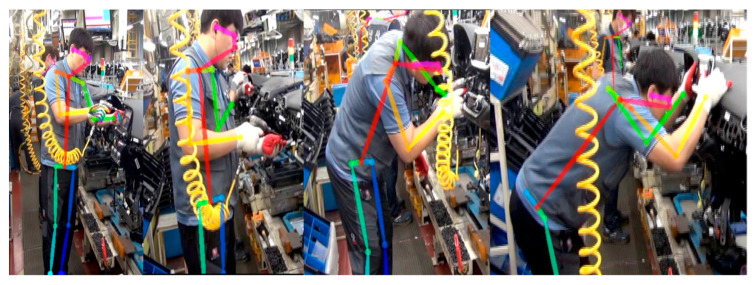
Results of body, foot, and hand pose estimation by OpenPose. Hand pose estimation problem due to assembly tool use with gloves on.

**Figure 4:**
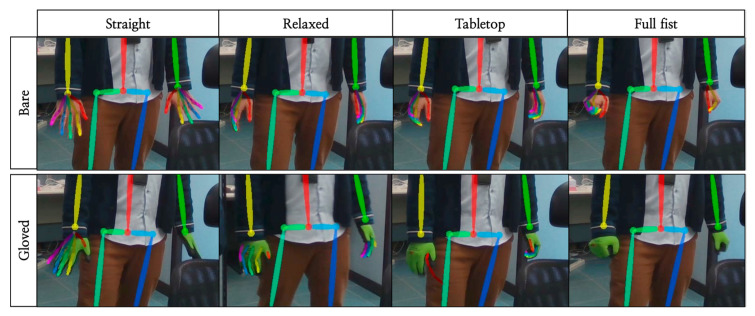
Comparison of OpenPose estimation results based on hand posture for bare and gloved hands.

We also used the postures of the cockpit module-assembly process workers in automobile manufacturing as inputs in OpenPose to estimate postures. The results indicate that the body pose estimation of the workers can be performed properly, as shown in Figure 2. However, problems arose when inferring the hand poses of the workers. As shown in Figure 3, OpenPose clearly estimates from the image representing the finger joint, but the estimation can be failed in continuous motion when holding the assembly tool in hand. We identified that this happens when workers wear gloves, and it causes a problem with hand-posture estimation. To verify this, we conducted an experiment in which images of bare hands and of gloves were captured, as shown in Figure 4. In the results, the same estimation problem occurred. The hand-posture estimation result was perfect, even when various postures were taken with bare hands, but the hand-posture estimation failed for full-fist hands wearing gloves. In particular, the hand-posture estimation was unstable in the state where the knuckles were bent.

We also examined the images of workers captured at the automobile cockpit module manufacturing process site and found that all workers were wearing gloves and in some cases aprons or sleeve protectors, depending on the process. In a manufacturing environment where wearing personal protective gear is unavoidable for the safety of workers depending on the characteristics of the workplace, it is essential to estimate the hand posture of workers wearing gloves when assessing the workers’ pose using RULA or REBA.

## 3. Building Extra Dataset

The open datasets used in pose-estimation models are produced by capturing images of professional actors acting in a studio or capturing images of postures taken in daily living. Thus, most key-point datasets do not contain the environmental information of our target manufacturing site and do not specialize in data for workers wearing various types of personal protective gear. Hence, in this study, we constructed a dataset with images of the automobile cockpit module manufacturing site to assess the poses of workers using all the pose assessment items required in the ergonomic musculoskeletal risk factor assessment tools.

**Figure 5:**
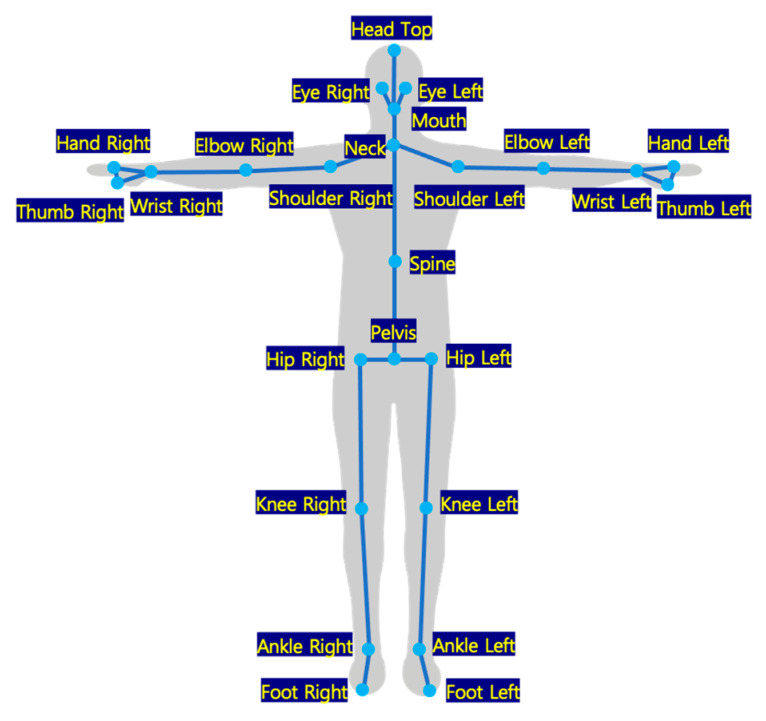
Definitions of joints.

The constructed dataset was based on the key-point structure of COCO [30]—a dataset typically used for training two-dimensional (2D) human pose-estimation models. We used the same index structure employed by the COCO dataset and added the required fingertip and tiptoe information. The dataset structure is shown in Figure 5.

First, we captured videos of workers performing unit tasks in the automobile cockpit module production plant. A total of 154 tasks were captured on video for more than nine hours, and all videos were recorded at 30 frames per second. Sixty of those tasks have a resolution of 720 × 480 and are compressed with MPEG-2, while the remaining 94 tasks are 1440 × 1080 and H.264 compressed. Then, to build the dataset from the videos, we used a web browser-based training data builder developed in-house.

**Figure 6:**
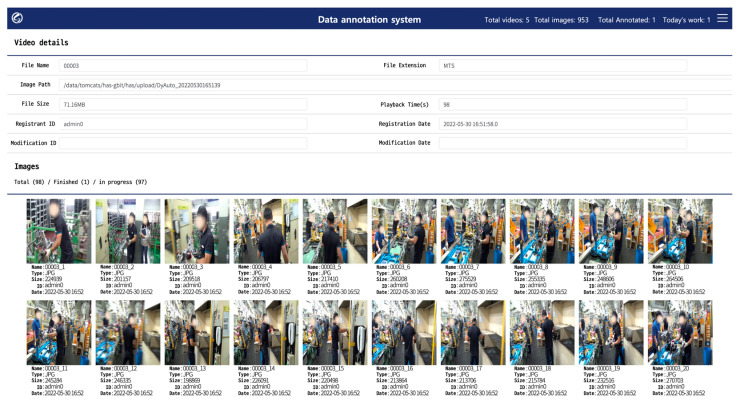
Images sampled at 1 s intervals by the web browser-based data annotation system.

**Figure 7:**
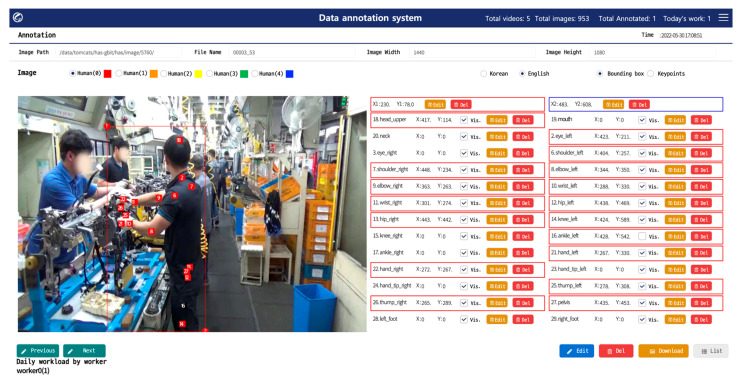
Graphic User Interface for creating annotated worker posture.

As shown in Figure 6, when the videos captured onsite are uploaded for each unit process; the system samples and saves images at one-second intervals. After that, an extracted image can be selected to annotate the bounding-box area and taggable joints for workers through the user interface, as illustrated in Figure 7. According to the annotator’s subjective judgment, joints that were invisible but could be clearly identified were recorded with the invisible attribute, and their information was added so that they could be used in training. To ensure that the data were not biased owing to the annotator’s personal judgment, an ID was assigned to each annotator, and the DB was designed to allow multiple annotators to tag joint information in the same image.

**Figure 8:**
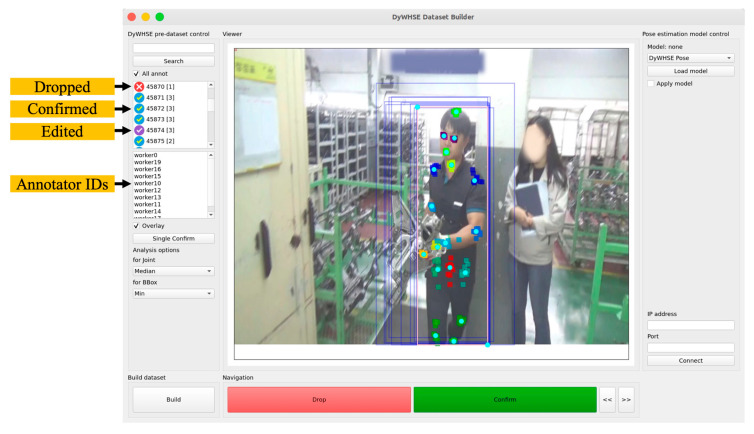
DyWHSE dataset builder.

Using this system, a number of annotators created 33,302 cases, and a DyWHSE dataset was finally built after validating the data using the DyWHSE dataset builder, as shown in Figure 8. The DyWHSE dataset builder overlays the joint information recorded by multiple annotators on the image so that the status of the data can be checked visually. As a tool with the function of confirming joint information created by annotators as a part of the dataset according to the inspector’s judgment, it provides coordinates highlighted in cyan for each joint as the recommended value based on the joint information recorded by the annotators. We developed the dataset builder with a simple interface consisting of two buttons—“Drop” and “Confirm”—so that the inspector could use this information to make a confirmation decision quickly.

If it is determined that the modification of the recorded information is unnecessary, the recommended value is recorded as-is in the dataset with the “Confirm” button. This is a method of obtaining a large amount of data in a limited time. If it is necessary to modify the coordinates of the recommended value, this can be done by dragging the joint with the mouse and then recording. Furthermore, a specific annotator selection function is provided so that only the data of highly reliable annotators can be used, at the discretion of the inspector.

**Figure 9:**
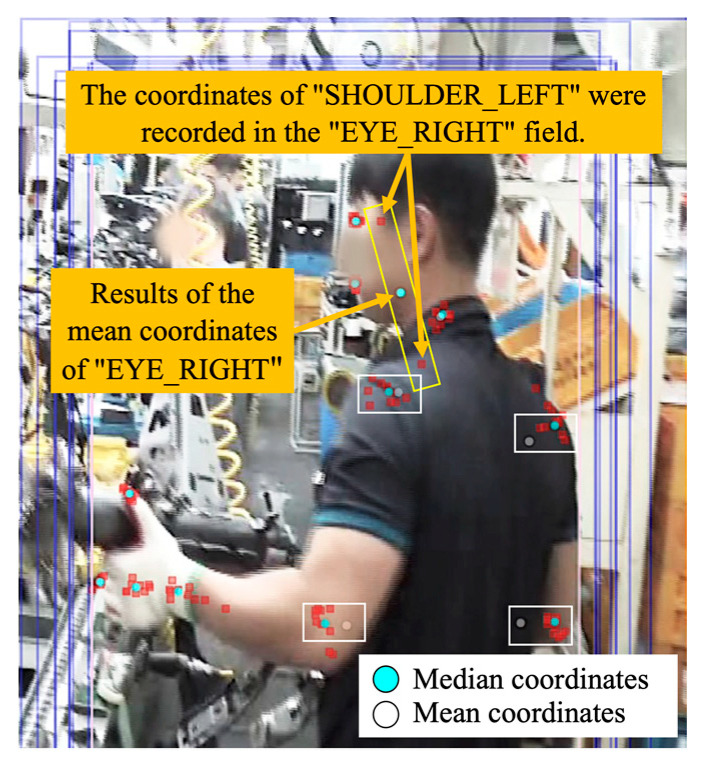
An error of the recommended value.

The recommended value refers to a function of the system that refines the joint location information recorded by multiple annotators and presents it on the image to help the inspector make a judgement. The coordinate information of the joint selected with the median value and the bounding box of the minimum region is obtained from the information recorded by multiple annotators. The mean value is provided as a recommended value to meaningfully use all the joint information recorded by the annotators; however, as shown in Figure 9, the recommended values are affected by the data of the annotator who mis-entered, among a number of annotators, the median value provided to the inspector.

**Figure 10:**
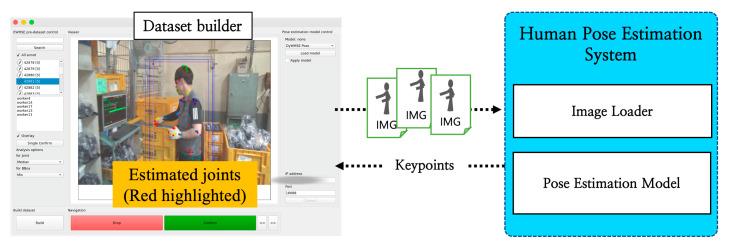
Block diagram of using an external pose-estimation model.

Another function of the dataset builder is to link the recommended value with the pose-estimation model, as presented in Figure 10, and provide the inspector with the information obtained from the model. This is a method of generating more data, but in the present study, we built the dataset using only the data recorded and inspected by humans, to increase the data accuracy.

## 4. Pose Model and Posture Assessment

Our objective is to develop an ergonomic WMSDs risk-assessment system that can be used continuously in manufacturing sites. To acquire images of assessment target work from the site quickly and easily, we used a single-view 3D human pose-estimation model for pose estimation and evaluated its performance by building an environment in which the DyWHSE dataset and existing datasets could be used together for training, as follows.

### 4.1. Modified Pose Model and Datasets

We used 3D-multi person pose estimation (3DMPPE) as a base model—a ResNet-152 [47]-based model proposed by Moon [23]—to validate the constructed DyWHSE dataset. This model comprises three modules: DetectNet [23], which estimates the bounding box of a person in an image; RootNet [23], which estimates the root coordinates centered on the camera; and PoseNet [23], which estimates relative poses according to the root. 3DMPPE used DetectNet, which was based on Mask-RCNN, and DetectNet required 120 ms to process a single frame in a single TitanX GPU. In the proposed system, YOLOv3 [48] was selected to detect workers as fast as possible. Among various versions of YOLO, YOLOv3-608 was chosen in the proposed system, requiring 51 ms to process a single frame in a single TitanX GPU. Furthermore, because we aim to assess the posture of a single worker, we changed the 3DMPPE’s multi-person pose estimation function to single person pose estimation (SPPE).

This model was trained by combining Human3.6M [32] and COCO, which are 3D and 2D datasets, respectively, to infer 17 joints in three dimensions. Human3.6M is a dataset built with 3D annotations that is widely used in human pose-estimation research. The actions of 11 professional actors were acquired indoors with a marker-based motion-capture system according to 15 scenarios, resulting in approximately 3.6 million images. We used two protocols of this dataset for the quantitative assessment of the model. In Protocol 1, subjects S1, S5, S6, S7, S8, and S9 were used for model training, and S11 was used for testing. In Protocol 2, S1, S5, S6, S7, and S8 were used for model training, and S9 and S11 were used for testing. In addition, we used the in-house-built 2D dataset DyWHSE as an extra dataset along with the K-pop dance video dataset [34], which is a 3D key-point dataset provided by AIHub [49].

### 4.2. Extra Datasets

The DyWHSE dataset specializes in environmental factors of the manufacturing process and was built with the goal of improving the hand-posture estimation of workers wearing protective gear such as gloves. However, because it was built on a 2D coordinate system and does not have the depth information of the newly added hand joints, the joint estimation results of 3DMPPE trained with this dataset cannot be used to assess the risk factors of musculoskeletal disorders. Therefore, to supplement this, we additionally used the K-pop dance video dataset for training. The K-pop dance video dataset is a 3D dataset that was constructed by motion capturing K-pop cover dances of professional dancers in studios. In addition, the DyWHSE dataset provides wrist, thumb, and hand keypoints for hand-posture estimation. For instance, the wrist angle can be calculated by using a plane consisting of wrist, thumb, and hand keypoints. However, the K-pop dance video dataset has keypoints, including wrist, thumb, and ring fingers. Thus, the mid-finger point is generated and used to match the hand keypoint in out experiment.

**Figure 11:**
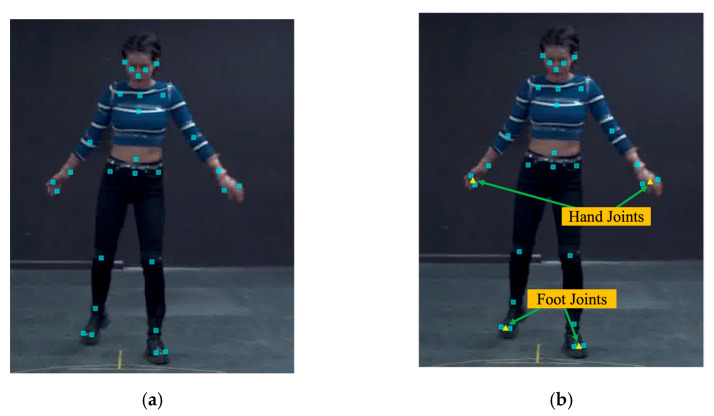
Extended K-pop dance video dataset of hand and foot joints: (**a**) K-pop dance video dataset’s defined joints; (**b**) generated joints that match the hands and feet of DyWHSE.

Figure 11 shows the defined joint and reproduced joints of the K-pop dance video dataset, and the 3D coordinate information of each hand and foot is provided as thumb–ring finger and big toe–little toe. To match DyWHSE and K-pop dance video datasets, we generated the joints of hand and foot by calculating the thumb–ring finger midpoint and the big toe–little toe midpoint; finally, we matched 25 joints used in training the model in the WMSDs risk-assessment system. The 3D datasets used to train DyWHSE-3DSPPE were Human3.6M, MuCo-3DHP [33], and K-pop dance video, and the 2D datasets were COCO, MPII [31], and the DyWHSE.

### 4.3. Posture Assessment

**Figure 12:**
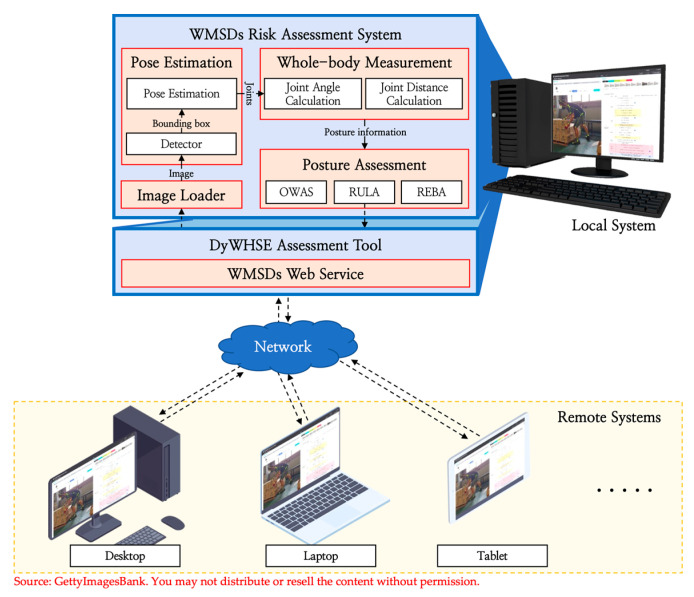
WMSDs risk-assessment system, which supports various computing environments.

In order to assess the posture of workers easily in various computing environments, we designed the WMSDs risk-assessment system with the structure shown in Figure 12. The user uploads the video of the worker via a web browser to the WMSDs web service, and when the analysis is requested, the system provides the result of the analysis. The following describes the internal operation of the system.

**Figure 13:**
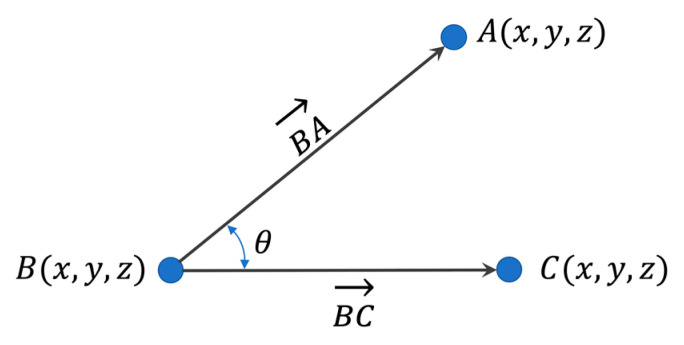
Calculate the angle of three joints.

The image loader reads the downloaded image, and the image is entered into the pose-estimation model after image preprocessing. The pose estimation module finds a person from the input image and estimates the joint within that area. According to the results, the whole-body measurement module calculates the angle and distance for the joints required in posture assessment. The whole-body measurement module calculates the angle and distance for all the joints required by the assessment module and preprocesses them to minimize redundant calculations in each posture-assessment tool. The joint angle is calculated by Equations (1)–(4) as three points in the 3D coordinate system, as shown in Figure 13, and the calculated distance information is used for the joint processing of the lower extremity state and the occluded image.

According to the situation on-site, occlusion can occur in a part of the body if taking a video of the whole body has been difficult. When posture is analyzed using the captured image, an incorrect joint coordinate is provided. This part is determined in an incorrect posture by using the length and angle value of the leg joint, providing the result value that only analyzes the upper body after assuming the posture legs and feet are supported.

(1) $$\vec{BA}=B-A$$

(2) $$\vec{BC}=B-C$$

(3) $$\vec{BC}\cdot \vec{BA}=\|\vec{BC}\|\|\vec{BA}\|\cos\theta$$

(4) $$\theta =\cos^{-1}\left(\frac{\vec{BC}\cdot \vec{BA}}{\left\|\vec{BC}\|\|\vec{BA}\right\|}\right)$$

The calculated angle and distance information are used as inputs in the WMSDs’ risk-assessment tools, and only the information of the joints that each assessment tool references is reconstructed and matched, according to the assessment tool’s rules. Finally, the assessment tool calculates the score for each part of the worker’s posture and determines the action level.

**Figure 14:**
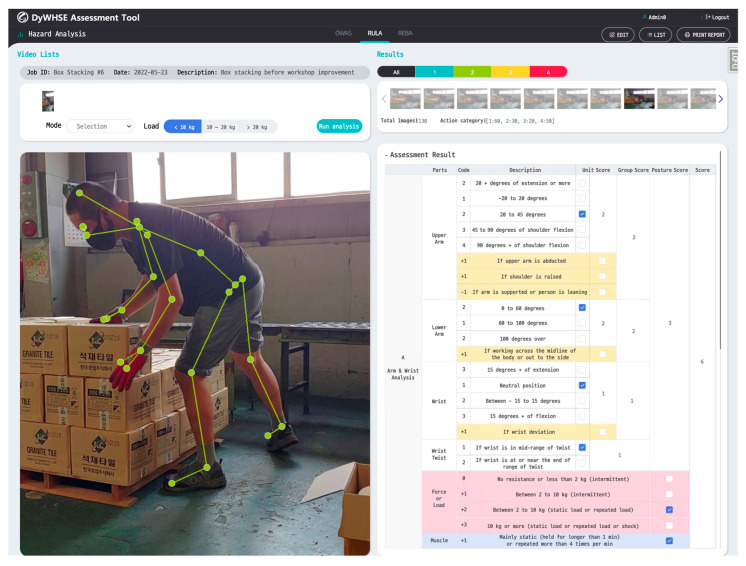
DyWHSE assessment tool.

We developed the DyWHSE assessment tool, which uses the web-based graphical user interface (GUI) to display the assessed worker’s pose information and the estimated joint information based on the WMSDs’ risk-assessment system. In this program, the user can modify the items in the table interface to recalculate the score for the errors in the pose-assessment model, and the program has the function of outputting the pose-assessment report. Furthermore, it provides an interface for a quick review of high-risk postures by providing a sorting function based on the scores of the analysis images, as shown in Figure 14.

## 5. Results

We used work-performing images of workers in the manufacturing industry to record the posture of one representative worker per image in the dataset. Through the validation, we constructed a 2D DyWHSE dataset for 15,849 images and expanded it to a DyWHSE-3DSPPE model to facilitate the inference of wrist poses in the 3DMPPE model by matching the joints in the hand area with the 3D K-pop dance video dataset.

To evaluate the performance of the model quantitatively and verify the quality of the dataset built in-house, we compared the performance of different models using the test protocols of Human3.6M, as shown in Table 2. As metrics for evaluating the similarity of postures, we used the mean per joint position error (MPJPE) [32] and procrustes analysis (PA) [50] MPJPE, which are widely used in 3D pose estimation. The unit for the MPJPE and PA-MPJPE was mm.

The MPJPE was calculated using Equation (5), i.e., the Euclidean distance between the ground truth and the inferred joint was evaluated as the mean error.

(5) $$MPJPE=\frac{1}{J}\overset{J}{\underset{j=1}{\sum }}\|Est_{j}-GT_{j}{\|}_{2}$$

Here, *j* represents the sample index, *J* represents the number of joints (*J* = 17), $Est_{j}$ represents the estimated joint, and $GT_{j}$ represents the ground truth. Because the structures of the reference dataset and the DyWHSE dataset were not identical, only the 17 matching joints between the two datasets are compared.

As indicated by Equation (6), PA-MPJPE is calculated using the ground truth after aligning the estimated joints using PA before the MPJPE calculation.

(6) $$PA-MPJPE=\frac{1}{J}\overset{J}{\underset{j=1}{\sum }}\|alignedEst_{j}-GT_{j}{\|}_{2}$$

Here, $alignedEst_{j}$ refers to the alignment of the estimated joints and shows the posture difference more purely than *MPJPE* by removing misalignments.

We employed the widely used Human3.6M dataset’s evaluation protocols to evaluate the model trained with DyWHSE. Under Protocol 1, six subjects—S1, S5, S6, S7, S8, and S9—were used for model training, and S11 was used for testing, as an PA-MPJPE assessment metric. Under Protocol 2, five subjects—S1, S5, S6, S7, and S8—were used for training, and two subjects—S9 and S11—were used for testing. They were used as MPJPE assessment metrics, and in accordance with previous works [23,24,25], every 5th frame and 64th frame in each video were used for training and testing, respectively. Furthermore, in accordance with [23,24,25], Human3.6M and MPII—a 2D dataset—were mixed (50:50), and the resulting dataset was used for training.

Using the foregoing evaluation method, we evaluated the DyWHSE-3DSPPE model and obtained the results shown in Table 2 and Table 3. When the model was trained by adding the DyWHSE dataset to the training dataset of 3DMPPE, the PA-MPJPE was 33.8 mm, and the MPJPE was 53.3 mm. In contrast, when the model was trained with all the datasets (COCO, Human3.6M, MPII, MuCo-3DHP, K-pop dance video, and DyWHSE), the PA-MPJPE was 32.5 mm, and the MPJPE was 46.3 mm.

**Figure 15:**
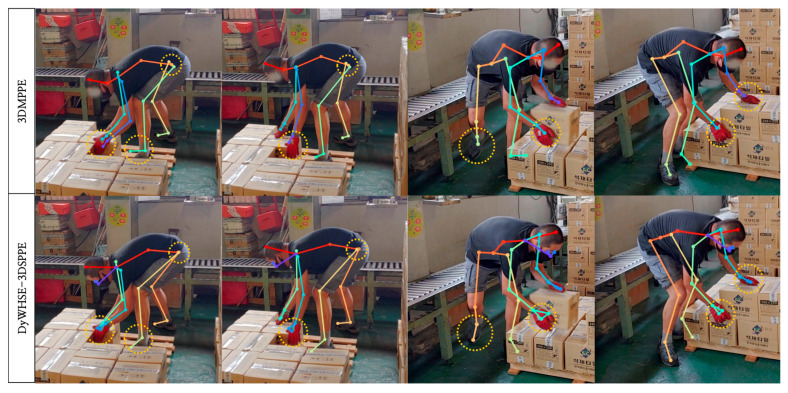
3DMPPE (MuCo-3DHP, COCO) and DyWHSE-3DSPPE (MuCo-3DHP, COCO) (DyWHSE, Human3.6M, MuCo-3DHP, MPII, COCO, and K-pop dance video) pose-estimation results for each model.

We used the 3DMPPE and DyWHSE-3DSPPE models and obtained the pose estimation of the worker, as shown in Figure 15. The 3DMPPE model provided only the joint information for the back of the hand, whereas the DyWHSE-3DSPPE model provided information for the thumb and back of the hand to judge wrist twists. The worker images used for the comparison were sampled at five frames per seconds (FPS) from work-performing images of a tile manufacturing company, which were not used when building the DyWHSE dataset.

**Figure 16:**
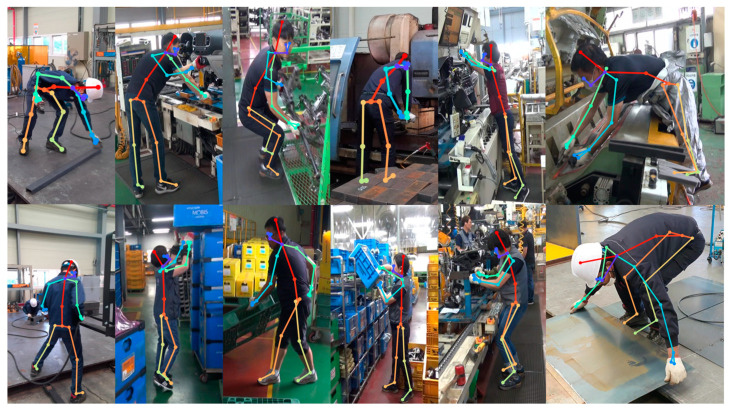
Image samples for RULA.

To evaluate the similarity between the proposed system and the expert assessment, the worker’s posture was inferred using DyWHSE-3DSPPE, and the posture score was extracted using the RULA assessment tool. The image used for assessment was obtained by acquiring work images of workers taking various working postures at three manufacturing plants and extracted at one FPS, and the sample images are shown in Figure 16.

The images used in the assessment were obtained from three manufacturing plants, and 200 images were provided to three ergonomics experts, who assessed the postures of the workers. Here, we used the common assessment results of 30 images as ground truths (GTs) and compared them with the system’s assessment results. For comparing the two systems, we performed a quantitative evaluation using Cohen’s kappa [52] coefficient to check whether the system’s pose-assessment result matched the GT. This coefficient represents the level of agreement between two evaluators and is defined as follows:

(7) $$\kappa =\left(p_{o}-p_{e}\right)/\left(1-p_{e}\right),$$

where $p_{o}$ represents the degree of agreement between the observers, and $p_{e}$ represents the probability of the results of two evaluators matching by coincidence.

We obtained results for the upper arm, lower arm, wrist, neck, trunk, and leg, as shown in Table 4. Cohen’s kappa coefficient ranges from –1 to 1, and –1 corresponds to a complete discrepancy and 1 corresponds to a complete agreement. The degree of agreement based on the kappa coefficient is presented in Table 5.

## 6. Discussion

Workers in manufacturing factories often wear gloves. By using OpenPose Whole Body Python API, hand-feature points cannot be detected when workers wear gloves, as shown in Figure 3 and Figure 4. That is why the DyWHSE data set is built in the proposed research, which includes factory workers with gloves.

According to our experimental results, DyWHSE-3DSPPE was verified to be effective for worker pose-estimation. When the DyWHSE dataset, which was built using images of workers producing automobile cockpit modules, was used with Human3.6M and MPII to train an RGB image-based 3D pose-estimation model, the results exhibited differences of 1.4 mm for PA-MPJPE and 1.1 mm for MPJPE, compared with the base model. This indicates that the DyWHSE dataset had equal precision to the datasets used for training. Furthermore, the COCO, MuCo-3DHP, and K-pop dance video datasets were combined and used to train the model, which resulted in a higher inference performance of 2.7 mm in the PA-MPJPE assessment and 8.1 mm in the MPJPE assessment. Although the DyWHSE dataset that we built was a 2D dataset with a size of approximately 15,000, it was used as an additional training dataset in the existing models, and as shown in Figure 15, the pose-estimation results of manufacturing process workers were reliable compared with those of the existing models.

However, for the wrist posture estimated by the DyWHSE-3DSPPE model, a quantitative performance evaluation could not be conducted, owing to the absence of an assessment dataset for matching the added joints. The Cohen’s kappa coefficient between the pose-assessment results of experts and RULA was also not of a high enough degree of agreement, but it is interesting that the k values ranged between 0.636 to 0.704 for the upper arm, lower arm, and trunk. The k value of 0.56 for the leg indicates that it is difficult to judge the worker’s leg support state only with the estimated joint information due to the floor structure of the site. In the case of the wrist, which was our target in this study, the degree of the agreement shows moderate performance. This joint has higher freedom of movement than other joints because the shape of the hand was affected by the use of tools.

Because of this problem, the WMSDs’ risk-assessment system cannot be fully automated. Nevertheless, it appears to be adequate for use as a support tool that can reduce the fatigue of experts that arises when they analyze the motions of numerous workers directly for WMSDs’ risk assessment. To this end, we provide a sorting interface to allow experts to focus on specific sections by setting a threshold for the assessed worker posture based on the action category. Furthermore, an intuitive interface is provided, where the assessment table can be modified directly so that the worker’s posture can be re-assessed depending on the judgment of an expert to cope with the inadequate performance of the pose-assessment model. In our assessment system, parameters such as the load information and repeated pose information, which cannot be received as inputs from the image, can be manually entered and reflected in the score calculation. We identified the strengths and limitations of the proposed method through a quantitative evaluation, and to overcome the limitations, we will expand and refine the datasets and improve the model in follow-up studies.

It is important to note that our contribution is not to improve the accuracy of the posture assessment with a posture assessment model or high-resolution sensors. Rather, we aimed to build a feasible system that collects video images of the worker and assesses work-related musculoskeletal disorders with a low-cost and easy-installation method in the manufacturing industrial environment. This is why the proposed system uses a single-view 3D pose-estimation model that can easily obtain the image despite lower accuracy than IMU or motion-capture system. We identified that existing solutions cannot include the wrist posture of workers and the high error of workers with gloves, although most workers wear gloves in the workplace. Unlike existing solutions, we built the training dataset of the wrist posture of the workers with gloves to enable the accurate pose assessment of workers in an industrial environment, as shown in Figure 15. Therefore, we can conclude that the contribution of this paper is to provide a feasible solution that can be applied to the manufacturing industrial environment, and to improve the pose assessment by adding the wrist posture with gloved workers.

It is also noticeable that the task at the workplace is recorded as a 2D image, and the 2D images are provided to the expert for the pose assessment, as with the traditional method. The proposed system also uses the same image but it applies a 3D joint estimation model in order to improve the joint-angle estimation and the posture assessment as well. We adopted Moon’s model [23] for the 3D joint estimation, and all the figures in the paper are the results of the projection of 3D posture estimation onto the 2D images. The proposed system can also convert the 3D posture estimation to the 2D image from the viewpoint of the left or right side.

## 7. Conclusions

We propose a DyWHSE-3DSPPE-based worker posture assessment system to support the wrist-posture assessment of the RULA assessment tool (an ergonomic work pose assessment tool) with a single-view 3D human pose-estimation model. We identified the problems associated with the difficulty of estimating wrist poses due to the gloves worn by workers in the manufacturing industry through OpenPose. To solve this problem, we developed a web browser-based data annotation system and DyWHSE dataset builder to build a dataset from images of manufacturing sites. Using these tools, we built a DyWHSE dataset with 2D coordinates containing 15,849 images of workers performing tasks in the automobile cockpit-module-producing process. Then, to compare the quality of this dataset with that of 3DMPPE (the base model), we trained the model using DyWHSE-3DSPPE, which extended the data to include hand joints. We evaluated the performance of the DyWHSE-3DSPPE model with the Human3.6M dataset. The results indicate an improvement of 2.7 mm in the PA-MPJPE compared with 3DMPPE. Furthermore, the MPJPE was 46.3 mm, corresponding to an improvement of 8.1 mm compared with that of 3DMPPE (54.4 mm). These results prove that the constructed dataset can improve the posture estimation performance, together with Keypoint datasets such as Human3.6M, MuCo-3DHP, MPII, and COCO. Finally, we used Cohen’s kappa coefficient to analyze the degree of agreement in the assessment results of 30 images of worker postures between our system and the RULA method used by experts. Although the performance of the proposed system is inferior to that of the expert assessment, the system can be employed to assist expert analysis using the DyWHSE assessment tool’s action-level-based pose classification interface and intuitive score-modification function.

## Figures and Tables

**Table 1:** Classification criteria for major working-posture-assessment tools.

Tool	Posture *	Parameter	Risk Levels
OWAS	Back (4), arms (3), and legs (7)	Force/load	4
RULA	Trunk (6), upper arm (6), lower arm (3), legs (2), neck (6), and wrist (4, + 2)	Force/load, muscle use	4
REBA	Trunk (5), upper arm (6), lower arm (2), legs (4), neck (3), and wrist (3)	Force/load, coupling, activity	5

* The number in parentheses refers to the number of postures classified in each assessment tool.

**Table 2:** Quantitative comparisons with the base model on the Human3.6M under Protocol 1 (PA-MPJPE). Best results are in bold.

Methods	Dire.	Disc.	Eat	Greet	Pho.	Pose	Purc.	Sit	SitD.	Smo.	Phot.	Wait	Walk	W.D.	W.T.	Avg.
**With ground truth information in inference time**																
**Sun [25] ^1^**	36.9	36.2	40.6	40.4	41.9	34.9	35.7	50.1	59.4	40.4	44.9	39.0	30.8	39.8	36.7	40.6
**Moon [23] ^1^**	31.0	30.6	39.9	35.5	34.8	30.2	32.1	35.0	43.8	35.7	37.6	30.1	24.6	35.7	29.3	34.0
**Ours ^2^**	29.8	30.4	38.9	35.7	34.0	29.4	30.4	33.0	41.1	35.0	37.3	29.8	24.1	35.4	27.8	33.1
**Ours ^3^**	30.0	**30.3**	38.6	35.2	33.5	31.7	30.8	33.7	41.4	34.1	37.8	30.0	24.5	36.2	28.1	33.3
**Ours ^4^**	**29.7**	**30.3**	**36.1**	**32.6**	**32.7**	**27.9**	**29.8**	**31.2**	**39.6**	**34.0**	**36.0**	**28.3**	**23.4**	**33.2**	**27.2**	**31.8**
**Without ground truth information in inference time**																
**Moon [23] ^1^**	32.5	31.5	41.5	36.7	36.3	31.9	33.2	36.5	44.4	36.7	38.7	31.2	25.6	37.1	30.5	35.2
**Ours ^2^**	29.6	**30.0**	39.1	35.0	35.2	29.9	31.0	36.4	41.8	37.3	37.7	29.4	24.1	35.5	28.4	33.8
**Ours ^3^**	**29.3**	**30.0**	39.0	35.6	34.6	30.7	30.7	36.7	42.5	36.1	38.3	29.0	24.4	35.7	28.6	33.7
**Ours ^4^**	29.5	30.6	**36.8**	**32.7**	**33.6**	**28.2**	**29.7**	**35.3**	**40.7**	**35.2**	**35.9**	**28.4**	**23.9**	**33.3**	**27.6**	**32.5**

^1^ Used Human3.6M and MPII synthetic data for training. ^2^ Used DyWHSE, Human3.6M, and MPII synthetic data for training. ^3^ Used DyWHSE, Human3.6M, and COCO synthetic data for training. ^4^ Used DyWHSE, Human3.6M, MuCo-3DHP, MPII, COCO, and K-pop dance video synthetic data for training.

**Table 3:** Quantitative comparisons with the base model on the Human3.6M under Protocol 2 (MPJPE). Best results are in bold.

Methods	Dire.	Disc.	Eat	Greet	Pho.	Pose	Purc.	Sit	SitD.	Smo.	Phot.	Wait	Walk	W.D.	W.T.	Avg.
**With ground truth information in inference time**																
**Sun [25] ^1^**	47.5	47.7	49.5	50.2	51.4	43.8	46.4	58.9	65.7	49.4	55.8	47.8	38.9	49.0	43.8	49.6
**Moon [23] ^1^**	50.5	55.7	50.1	51.7	53.9	46.8	50.0	61.9	68.0	52.5	55.9	49.9	41.8	56.1	46.9	53.3
**Ours ^2^**	47.0	50.7	47.0	49.9	49.3	44.8	47.3	58.1	60.9	49.0	53.8	47.0	38.7	50.8	43.6	49.5
**Ours ^3^**	45.4	52.8	49.5	49.3	51.7	45.1	50.1	64.9	68.8	51.7	54.4	48.8	40.4	55.5	45.8	52.0
**Ours ^4^**	**41.1**	**45.9**	**44.9**	**44.1**	**47.5**	**39.2**	**42.8**	**55.4**	**59.1**	**46.0**	**50.4**	**41.9**	**37.3**	**48.2**	**41.8**	**46.1**
**Without ground truth information in inference time**																
**Rogez [51] ^5^**	55.9	60.0	64.5	56.3	67.4	71.8	55.1	55.3	84.8	90.7	67.9	57.5	47.8	63.3	54.6	63.5
**Moon [23] ^1^**	51.5	56.8	51.2	52.2	55.2	47.7	50.9	63.3	69.9	54.2	57.4	50.4	42.5	57.5	47.7	54.4
**Ours ^2^**	48.7	55.8	49.3	50.9	56.1	47.1	48.5	63.2	68.6	52.6	57.4	50.0	39.7	55.2	45.2	53.3
**Ours ^3^**	49.6	55.0	49.9	51.2	54.3	47.5	50.1	63.5	63.5	53.2	58	49.1	40.9	54.7	45.3	53.0
**Ours ^4^**	**42.4**	**47.7**	**43.8**	**44.1**	**48.9**	**40.9**	**43.7**	**54.7**	**56.3**	**47.3**	**49.1**	**42.6**	**36.3**	**47.1**	**41.2**	**46.3**

^1^ Used Human3.6M and MPII synthetic data for training. ^2^ Used DyWHSE, Human3.6M, and MPII synthetic data for training. ^3^ Used DyWHSE, Human3.6M, and COCO synthetic data for training. ^4^ Used DyWHSE, Human3.6M, MuCo-3DHP, MPII, COCO, and K-pop dance video synthetic data for training. ^5^ Used Human3.6M, MPII, COCO, and CMU Mocap synthetic data for training.

**Table 4:** Comparison between the results of the system and expert assessments.

	Upper Arm	Lower Arm	Wrist	Neck	Trunk	Leg
**K**	0.698	0.636	0.456	0.587	0.704	0.516

**Table 5:** Strength of agreement using the kappa coefficient.

Kappa Range	Strength of Agreement
κ≤0	Poor
0.01≤κ≤0.2	Slight
0.21≤κ≤0.4	Fair
0.41≤κ≤0.6	Moderate
0.61≤κ≤0.8	Substantial
0.81≤κ≤1	Almost perfect

## Data Availability

The DyWHSE Dataset built up during the current study will not be publicly available.
